# Comparison of Somatic Mutation Profiles Between Formalin-Fixed Paraffin Embedded Tissues and Plasma Cell-Free DNA from Ovarian Cancer Patients Before and After Surgery

**DOI:** 10.1089/biores.2019.0031

**Published:** 2020-03-13

**Authors:** Marianna Jagelkova, Katarina Zelinova, Zuzana Laucekova, Martina Bobrovska, Zuzana Dankova, Marian Grendar, Karol Dokus

**Affiliations:** ^1^ Division of Oncology, Biomedical Center Martin, Jessenius Faculty of Medicine in Martin, Comenius University in Bratislava (JFM CU), Martin, Slovakia.; ^2^ Clinic of Gynecology and Obstetrics, Martin University Hospital (MUH) and JFM CU, Martin, Slovakia.; ^3^ Department of Pathological Anatomy, MUH and JFM CU, Martin, Slovakia.; ^4^ Department of Bioinformatics, Biomedical Center Martin JFM CU, Martin, Slovakia.; ^5^ 2nd Department of Gynecology and Obstetrics, Faculty Hospital with Policlinic of F. D. Roosevelt, Slovak Medical University, Banska Bystrica, Slovakia.

**Keywords:** circulating tumor DNA, gene panel sequencing, mutation profile, ovarian carcinoma

## Abstract

Ovarian carcinogenesis can be induced by a large number of somatic gene mutations. Circulating tumor DNA (ctDNA) released into peripheral blood can provide insights into the genomic landscape of cancer cells and monitor their dynamics. Our aim was to detect and compare the genetic profiles in tumor tissue and plasma before and after tumor resection in ovarian cancer patients. All three samples were collected from each patient. In this study, we used a commercial cancer panel to identify somatic mutations in 26 genes in seven selected patients through next-generation sequencing on the Illumina platform. Overall, 16 variants with pathogenic effect were identified in the *TP53*, *PIK3CA*, *PTEN*, *APC*, *NRAS*, *KRAS*, *GNAS*, and *MET* genes involved in important signaling pathways. The genetic alterations found in the presurgical plasma in six of seven ovarian cancer patients were no longer present in the plasma after tumor surgical removal. Identical variants in formalin-fixed paraffin embedded (FFPE) tissues and preoperative plasma specimens were observed in only two cases. These findings suggest that the detected presurgical pathogenic variants absent in postsurgery plasma are associated with the primary ovarian tumor. Finally, the low-identified concordance between FFPE and plasma can be due to various factors, but most likely to high tumor heterogeneity and low ctDNA level.

## Introduction

Ovarian cancer is the third most common female malignancy and the second global cause of cancer-related deaths from gynecological cancers. The International Agency for Research on Cancer also reports that this is the most lethal female genital tract oncology disease in Europe.^1^ Moreover, ovarian cancer is an aggressive disease, and its high biological and genetic heterogeneity is caused by multiple genetic and epigenetic changes in genes participating in important cellular signaling pathways. The following signaling pathways are involved in ovarian carcinogenesis; (1) p53; (2) PI3K/PTEN; (3) Ras/Raf/MAPK and (4) Wnt/ß-cat signaling pathway. Deregulated and altered pathways can lead to induced cell proliferation, apoptotic inhibition, increased motility, adhesion, invasion, and angiogenesis.^2–6^ In addition, late diagnosis, malignant progression, poor prognosis, and chemoresistance are major problems in many ovarian cancers.^6,7^ Tumor tissue biopsy analysis is most generally the standard diagnostic procedure.

The discovery that cell-free DNA (cfDNA) is present in the blood has provided significant benefits in clinical and experimental medicine. This cfDNA is double-stranded, fragmented extracellular DNA released into the blood circulation from cells through active secretion or apoptosis.^8^ Many researchers working with cfDNA have demonstrated that cancer-related genetic changes such as point gene mutations, copy number variations (CNVs), loss of heterozygosity, and chromosomal aberrations can be detected in the circulating DNA fragments released from tumor cells. This may reflect the genomic landscape of the primary tumor and metastases.^9–14^ Circulating tumor DNA (ctDNA) originate from apoptotic or necrotic cancer cells, perhaps even from active cellular secretion, and its fragment size is most commonly less than 180 bp.^15–20^ Moreover, ctDNA usually constitutes a small fraction (<20%) of total cfDNA concentration that is generally low. While its levels correlate with disease grading, staging, and metastasis, this can vary among patients.^9,21–26^

In addition to ctDNAs, circulating nucleic RNAs and miRNAs, circulating tumor cells, and extracellular vesicles represent a new approach called “Liquid biopsy” that allow to analyze the genetic and epigenetic profiles from many body fluids, especially blood serum and plasma, saliva, urine, and so on.^26,27^ Liquid biopsy can provide benefits when standard tissue biopsy cannot be performed due to difficulty in accessing tissue specimens, and it is equally beneficial in monitoring tumor heterogeneity, the emergence of drug resistance, and the determination of minimal residual disease following surgery and therapy.^28,29^

Highly sensitive and specific approaches are required for accurate and reliable genetic analyses because of limited plasma ctDNA. Next-generation sequencing (NGS) presents high-throughput technology, which enables analysis of a large number of different DNA sequences in parallel. These sequencing methods can detect various genomic alterations, including single and multiple nucleotide variations (SNV/MNV), insertions (ins), deletions (del), and CNVs.^30^

The aim of our study is to detect somatic mutations in 26 genes in ovarian tumors and monitor changes in the mutational profiles before and after surgery using liquid biopsy. We also compare the genetic variants found in plasma with the tumor tissue specimens from the same patients.

## Materials and Methods

### Patients and samples

This study examined patients with ovarian cancer diagnosed by histology. It was approved by The Ethics Committee (EC 1525/2014), implemented in accordance with the Declaration of Helsinki, and all patients provided written informed consent for participation in the study. Formalin-fixed paraffin embedded (FFPE) tissues and blood samples taken before surgery and the second day after surgery were used for the genetic analysis. Here, 10 mL of blood from each participant was collected in K_3_EDTA tubes at the Clinic of Gynecology and Obstetrics at the Jessenius Faculty of Medicine in Martin, the Comenius University in Bratislava and University Hospital in Martin. Plasma was separated within 2 h of blood collection by two-step centrifugation (2,200 *g*, 8 min, 4°C; 20,000 *g*, 8 min, 4°C). The biopsy material was obtained at the Department of Pathological Anatomy at the Jessenius Faculty of Medicine in Martin and the two University sites mentioned above.

### Extraction and quantification of DNA

DNA from FFPE samples was extracted with the blackPREP FFPE DNA kit (Analytic Jena, Germany) and cfDNA isolation was performed from 3 to 4 mL of plasma using the QIAamp DSP Virus kit (QIAGEN, Germany) according to protocol. The isolated cfDNA was concentrated by complete drying at 37°C in the CentriVap Concentrator (LABCONCO) and then by dissolution in 40 μL of ultrapurified water. The concentration of DNA was measured using the Qubit BR dsDNA Assay Kit or the Qubit HS dsDNA Assay Kit (Life Technologies) on the Qubit 2.0 Fluorometer (Invitrogen). DNA quality from the FFPE blocks was then determined by the KAPA SYBR Fast Master Mix Universal Kit (KAPA Biosystems) in the 7500 Fast Real-Time Polymerase Chain Reaction (PCR) System (Applied Biosystems). Preparation of the PCR and the PCR program was performed according to the TruSight Tumor (TST) 26 protocol (Illumina). The total DNA input was calculated based on the obtained ΔCT values.

### Preparation of the DNA library and sequencing

The recommended DNA input was from 30 to 300 ng dependent on DNA specimen quality. The sequencing DNA libraries were then prepared using the TST 26 Kit (Illumina) which enables the detection of somatic variants with frequency below 5% in 26 genes (Table 1). The quality of DNA libraries was assessed by the Agilent High Sensitivity DNA Kit (Agilent Technologies, Germany) on the 2100 Bioanalyzer instrument (Agilent Technologies) and their quantity was determined by the Qubit 2.0 Fluorometer (Invitrogen). The DNA libraries were then diluted to the final molarity (4 nM), denatured, pooled in one reaction, and run on the MiSeq platform using the MiSeq Reagent Kit v2 over 300 cycles (Illumina). Each step was performed according to the manufacturer's instructions and the number of specimens in each sequencing run was limited to four paired samples.

**Table 1.** TruSight Tumor 26 Gene List (Illumina**)**

*AKT1*	*EGFR*	*GNAS*	*NRAS*	*STK11*
*ALK*	*ERBB2*	*KIT*	*PDGFRA*	*TP53*
*APC*	*FBXW7*	*KRAS*	*PIK3CA*	
*BRAF*	*FGFR2*	*MAP2K1*	*PTEN*	
*CDH1*	*FOXL2*	*MET*	*SMAD4*	
*CTNNB1*	*GNAQ*	*MSH6*	*SRC*	

### Bioinformatic analysis

Resultant sequencing reads in FASTAQ format were aligned to the Human reference genome hg19 and final variant call files (vcf) were generated by MiSeq Reporter Software (v2.6). These vcf data were then processed through the bioinformatic online tool—Variant Effect Predictor (vs95) (https://www.ensembl.org/info/docs/tools/vep/index.html). Individual genetic variants were filtered based on the following criteria; variant call quality = 100 and the base coverage of each region >1000 × . These were then evaluated using the external COSMIC (vs88) somatic mutations database (https://cancer.sanger.ac.uk/cosmic) and the Varsome (vs6.7) free variant data discovery tool (https://varsome.com). Finally, variant pathogenicity was classified according to the American College of Medical Genetics and Genomic guidelines (ACMG).^31^

## Results

**FIG. 1.**
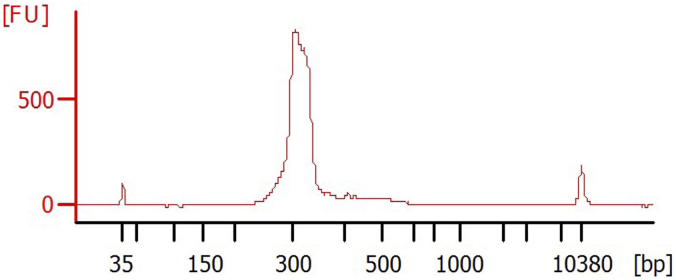
The Agilent 2100 Bioanalyzer electropherogram shows the purity and size of the DNA library fragments. The suitable size ranges from 300 to 330 bp and the peak intensity depends on the amount of amplified DNA fragments. The peaks at 35 and 10,380 bp delineate the lower and upper internal control markers used to align the ladder with the sample. FU, fluorescence unit.

Table 2 lists the baseline characteristics for the seven 54–78 years old postmenopausal women selected for this study. In this study, we analyzed three specimens (FFPE tissue and plasma sample before and after tumor resection) from each patient. The amount of DNA extracted from FFPE ranged from 68.4 to 760 ng/μL and the entire range of cfDNA level was 2.54 to 9.68 ng/μL, with average 4.858 ± 1.86 ng/μL. We then prepared all appropriately sized DNA libraries for DNA sequencing. Figure 1 herein highlights the DNA library quality.

**Table 2.** Baseline Clinical Characteristics of Patients Diagnosed with Malignant Ovarian Cancer

Patient	Age at diagnosis	Histology	Grade	TNM
1	63	Serous adenocarcinoma	G3	pT3c pNx pMx
2	54	Serous carcinoma	G3	ypTx pNx pM1
3	63	Seromucinous carcinoma	G2	pT1c2 pNx pMx L0 V1 Pn0
4	55	Serous carcinoma	G3	pT3c pN1 pMx, L1 V1 Pn0.
5	78	Serous adenocarcinoma	G1	pT3c pNx pMx, L1 Vx Pnx
6	59	Serous carcinoma	G3	—
7	54	Serous carcinoma	G3	pT3b pN0 pMx, L1 V0 Pn0

—, data not available.

We identified 16 pathogenic or likely pathogenic variants in this cohort. These included SNVs, deletions, and duplications in eight genes. The identified 16 variants comprised 7 missense, 3 nonsense, 3 frameshift, 1 in frame insertion, 1 splice donor, and 1 splice acceptor mutation. Table 3 shows the genetic alterations detected with variant allele frequency (VAF) >3%.

**Table 3.** Variants Identified by Next-Generation Sequencing Gene Panel in Seven Women with Diagnosed Ovarian Cancer

Patient	DNA sample	Chrom:position	Gene	Coding sequence	Consequence	Coverage	VAF %
1	Before surgery	10:89725045	*PTEN*	c.1028T>A	Missense, splice region	12,092	3.92
		20:57480480	*GNAS*	c.430G>T	Missense	16,569	3.23
	After surgery						
	FFPE	17:7578535	*TP53*	c.393_395dupCAA	Inframe insertion	6,644	61.63
2	Before surgery	17:7578263	*TP53*	c.586C>T	Nonsense	21,353	3.77
		5:112175634	*APC*	c.4344delC	Frameshift	22,465	3.08
	After surgery						
	FFPE	1:115256529	*NRAS*	c.182A>G	Missense	52,079	40.95
3	Before surgery	10:89624307	*PTEN*	c.79 + 2T>C	Splice donor variant	10,374	3.03
	After surgery						
	FFPE	3:178936091	*PIK3CA*	c.1633G>A	Missense	30,428	43.71
4	Before surgery						
	After surgery	17:7579585	*TP53*	c.102dupC	Frameshift	24,156	3.11
	FFPE	17:7576928	*TP53*	c.920-2delA	Splice acceptor variant	7,806	18.01
		17:7577538	*TP53*	c.743G>A	Missense	17,449	27.59
5	Before surgery	12:25398284	*KRAS*	c.35G>T	Missense	14,851	3.50
		7:116381017	*MET*	c.1639C>T	Nonsense, stop codon	24,756	3.68
	After surgery	1:115258744	*NRAS*	c.38G>A	Missense	11,422	5.10
	FFPE	12:25398284	*KRAS*	c.35G>T	Missense	25,274	23.87
6	Before surgery	17:7577538	*TP53*	c.743G>A	Missense	21,209	4.28
		5:112175761	*APC*	c.4473dupT	Frameshift	11,694	3.22
	After surgery						
	FFPE	17:7577538	*TP53*	c.743G>A	Missense	91,332	74.19
7	Before surgery	3:178951919	*PIK3CA*	c.2974C>T	Nonsense, stop codon	25,473	4.56
	After surgery						
	FFPE						

del, deletion; dup, duplication; FFPE, formalin-fixed paraffin embedded tissue; VAF, variant allele frequency.

The COSMIC database confirmed that all identified variants except the *PTEN* c.1028T>A, *GNAS* c.430G>T, and *TP53* c.393_395dup found in Patient 1 were somatic. We consider that these genetic changes are somatic because they were not present in cfDNA after tumor resection and had low VAF—generally characteristic for the somatic mutations.^32,33^ The most mutated variants were observed in the *TP53* gene in the high-grade serous ovarian carcinoma (HGSOC) patients.

In addition, all somatic pathogenic variants found in the presurgical ctDNA samples in six of seven patients were not identified in the plasma after tumor resection. However, we found a postsurgery somatic *NRAS* mutation in the plasma from Patient 5, which was not detected in the ctDNA before tumor removal. The seventh of the above patients, Patient 4, had one *TP53* mutation in the postoperative sample, although no somatic pathogenic variants were identified in the presurgery plasma.

All analyzed FFPE samples were derived from the primary tumor with the exception of one metastatic FFPE tissue in Patient 2 because the primary tumor could not be assessed by histology. The same pathogenic variants (*KRAS* c.35G>T and *TP53* c.743G>A) found in FFPE specimens from Patients 5 and 6 were detected in their ctDNA samples before surgery, but were not present in the plasma after successful tumor removal.

The Clinical Interpretation of Variants in Cancer database (CIViC) defines that the c.35G>T variant results in the loss of GTPase activity and this leads to a constitutively active form of the *KRAS* gene and the c.743G>A variant has worse overall survival and increased invasive behavior. Also, the *PTEN* c.1028T>A variant identified in Patient 3 may confer resistance to EGFR inhibitors such as CETUXIMAB. In addition, no correlation between FFPE of primary tumor/metastasis and preoperative ctDNA variants were observed in the remaining five patients. This discrepancy found in the mutational profiles of the same patient could be due to high tumor heterogeneity or very low VAF.

## Discussion

Our study analyzed the mutational profiles of tumor tissues and cfDNA and this revealed pathogenic variants in the eight *TP53*, *PIK3CA*, *PTEN*, *APC*, *NRAS*, *KRAS*, *GNAS*, and *MET* genes in patients with ovarian carcinoma. Most patients were diagnosed with HGSOC and their *TP53* gene was the most frequently mutated. This is supported by the OncoMap and The Cancer Genome Atlas (TCGA) large-scale studies.^4,34^

In this study, we were able to identify somatic mutations with VAF from 3.03% to 5.10% in cfDNA and 18.01% to 74.19% in FFPE tissues. This agrees with most recent study results that reveal that the TST 26 panel can detect somatic alterations with VAF ≥3%.^35^ However, Giardina et al.^36^ demonstrated a detectable VAF less than 3% with this gene panel. Several studies focused on the comparison of pre- and postsurgical ctDNA reported decreased frequency of mutated variants after tumor resection in various cancers. Sun et al.^37^ demonstrated that the postoperative mutation frequency decreased compared with preoperative ctDNA variants matched those were detected in tumor tissue in the majority of patients; Chan et al.^38^ detected tumor-associated copy number aberrations that disappeared almost completely in plasma samples 1 week after tumor removal; Harris et al.^24^ found somatic chromosomal rearrangements in plasma samples after surgery in only patients with detectable disease, while no aberrations in those without continued disease; and Ng et al.^39^ observed almost no primary tumor-specific mutations in the postsurgical ctDNA that were present in the plasma samples before surgery in colorectal cancer patients. Further researchers also demonstrated that the frequency of preoperative mutated variants identified in lung cancer by NGS approaches was significantly decreased or completely disappeared within 2 days of surgery.^40,41^ This supports our results that the preoperative plasma variants were not detected in plasma the second day after tumor elimination in all instances except for two patients who had one postsurgical variant, but none presurgically. The presence of these postoperative mutations is likely due to minimal residual disease or metastasis.^42^

Comparison of FFPE and preoperative plasma specimens revealed large differences in identified variants. The same mutations in the presurgical plasma and primary tumor were found in only two cases. Some studies reported 68–100% concordance of mutations detected in plasma and tumor tissues.^10,11,43^ Our gene panel sequencing method differed vastly to the number of genes examined by the above-mentioned researchers. Phallen et al.^10^ analyzed 58 genes in various cancers, Oikkonen et al.^11^ investigated over 500 genes in ovarian cancer patients with different NGS approaches and Kim et al.^43^ examined genetic changes in only one gene using different mutation analysis. In contrast, other NGS analysis of BRCA1/2 mutations in ovarian cancer demonstrated 100% concordance between the tumor and ctDNA germline variants, but this was not observed in the case of somatic variants, indicating the intratumor heterogeneity.^44^

The main limitation of our study was a small sample size. Possible reasons for the discordance between tumor and plasma variants include the following: (1) high tumor heterogeneity^45^; (2) low ctDNA level/VAF^46^; (3) the quality of sample and sequencing run; and (4) low clustering. The cfDNA concentrations were low and the ctDNA fraction was not measured. Although the cluster density was lower in the 500 to 600 K/mm^2^, range for three presurgical plasma samples and quality score was less than 80%, for two FFPE specimens, all other sequencing runs had over 92% quality and cluster density of ∼900 K/mm^2^.*

In summary, all identified somatic pathogenic preoperative mutations were absent in the patient plasma after successful tumor resection, and this indicates that the detected presurgical variants are associated with the primary ovarian tumor. Moreover, the observed discrepancy between tumor and plasma variants is likely due to high tumor heterogeneity, low ctDNA level, or degraded cfDNA samples with poor quality. Therefore, we think that ctDNA analysis has the potential in identifying tumor heterogeneity and monitoring patients in postsurgical treatment, but larger patient cohort examination is required to optimize the analysis. This especially applies to a larger input of plasma volume for cfDNA extraction which will then increase detectable ctDNA levels.

## Conclusion

The ctDNA analysis cannot completely replace the conventional tumor biopsy diagnosis of oncological diseases and certain limitations still preclude the liquid biopsy implementation in clinical practice. However, we assume that ctDNA can contribute to detecting tumor heterogeneity, improving diagnosis and monitoring patient postsurgical treatment. This is achievable by investigating larger patient cohorts to increase the specificity and sensitivity of ctDNA analysis to an appropriate level for improved ovarian and other cancer diagnosis and treatment.

## Abbreviations Used

ACMG: American College of Medical Genetics and Genomic
cfDNA: cell-free DNA
ctDNA: circulating tumor DNA
CIViC: Clinical Interpretations of Variants in Cancer
CNV: copy number variations
Del: deletion
Dup: duplication
FFPE: formalin-fixed paraffin embedded
FU: fluorescence unit
G: grade
HGSOC: high-grade serous ovarian carcinoma
Ins: insertion
MNV: multiple nucleotide variations
NGS: next-generation sequencing
SNV: single-nucleotide variations
TST: TruSight Tumor
VAF: variant allele frequency
vcf: variant call file
PCR: polymerase chain reaction
TCGA: The Cancer Genome Atlas

## Notes

^*^ Recommended cluster density values of 1000–1200 K/mm^2^ advised on the site http://emea.support.illumina.com/bulletins/2016/10/cluster-density-guidelines-for-illumina-sequencing-platforms-.html?langsel=/sk/.
